# Comparative Analysis of the Combined Effects of Different Water and Phosphate Levels on Growth and Biological Nitrogen Fixation of Nine Cowpea Varieties

**DOI:** 10.3389/fpls.2017.02111

**Published:** 2017-12-19

**Authors:** Martin Jemo, Saad Sulieman, Faouzi Bekkaoui, Oluwatosin A. K. Olomide, Abeer Hashem, Elsayed Fathi Abd_Allah, Abdulaziz A. Alqarawi, Lam-Son Phan Tran

**Affiliations:** ^1^AgroBiosciences Division, Mohammed VI Polytechnic University (UM6P), Ben Guerir, Morocco; ^2^Office Chérifien des Phosphates (OCP)-Africa, Casablanca, Morocco; ^3^Department of Agronomy, Faculty of Agriculture, University of Khartoum, Shambat, Sudan; ^4^Department of Agronomy, University of Ibadan, Ibadan, Nigeria; ^5^Botany and Microbiology Department, College of Science, King Saud University, Riyadh, Saudi Arabia; ^6^Mycology and Plant Disease Survey Department, Plant Pathology Research Institute, ARC, Giza, Egypt; ^7^Plant Production Department, College of Food and Agricultural Sciences, King Saud University, Riyadh, Saudi Arabia; ^8^Institute of Research and Development, Duy Tan University, Da Nang, Vietnam; ^9^Signalling Pathway Research Unit, RIKEN Center for Sustainable Resource Science, Yokohama, Japan

**Keywords:** biological nitrogen fixation potential, cowpea varieties, water-deficit damage, dry savanna of Nigeria, phosphate deficiency

## Abstract

Water deficit and phosphate (Pi) deficiency adversely affect growth and biological nitrogen fixation (BNF) of legume crops. In this study, we examined the impact of interaction between soil water conditions and available soil-Pi levels on growth, nodule development and BNF potential of nine cowpea varieties grown on dry savanna soils. In our experimental design, soils with different available soil-Pi levels, i.e., low, moderate, and high soil-Pi levels, collected from various farming fields were used to grow nine cowpea varieties under well-watered and water-deficit conditions. Significant and severe water deficit-damaging effects on BNF, nodulation, growth, levels of plant-nitrogen (N) and -phosphorus (P), as well as shoot relative water content and chlorophyll content of cowpea plants were observed. Under well-watered and high available soil-Pi conditions, cowpea varieties IT07K-304-9 and Dan'Ila exhibited significantly higher BNF potential and dry biomass, as well as plant-N and -P contents compared with other tested ones. Significant genotypic variations among the cowpeas were recorded under low available soil-Pi and water-deficit conditions in terms of the BNF potential. Principal component (PC) analysis revealed that varieties IT04K-339-1, IT07K-188-49, IT07K-304-9, and IT04K-405-5 were associated with PC1, which was better explained by performance for nodulation, plant biomass, plant-N, plant-P, and BNF potential under the combined stress of water deficit and Pi deficiency, thereby offering prospects for development of varieties with high growth and BNF traits that are adaptive to such stress conditions in the region. On another hand, variety Dan'Ila was significantly related to PC2 that was highly explained by the plant shoot/root ratio and chlorophyll content, suggesting the existence of physiological and morphological adjustments to cope with water deficit and Pi deficiency for this particular variety. Additionally, increases in soil-Pi availability led to significant reductions of water-deficit damage on dry biomass, plant-N and -P contents, and BNF potential of cowpea varieties. This finding suggests that integrated nutrient management strategies that allow farmers to access to Pi-based fertilizers may help reduce the damage of adverse water deficit and Pi deficiency caused to cowpea crop in the regions, where soils are predominantly Pi-deficient and drought-prone.

## Introduction

The biological nitrogen fixation (BNF) process occurring in specialized plant structure called nodules confers to leguminous crops the ability to convert the atmospheric dinitrogen (N_2_) via rhizobia for usable nitrogen (N) to satisfy their own and other plants' N-source demand (Giller, 2001; Udvardi and Poole, 2013). Through this process, legumes have been known to play vital roles in cropping cycles for the benefit of millions of small farmers (Graham and Vance, 2003; Langyintuo et al., 2003; Sanginga, 2003; Crews and Peoples, 2004). However, under their growing environment, the BNF process is largely hampered due to abiotic stresses, such as drought and/or low availability of phosphate (Pi) [a consumable form of phosphorus (P) for plants] in soils, causing diminution of BNF and yield loss (Tran and Nguyen, 2009; Thao and Tran, 2012; Sulieman and Tran, 2013; Sulieman et al., 2013; Aranjuelo et al., 2014; Nasr Esfahani et al., 2014). These two abiotic stresses, drought and Pi deficiency, either alone or together, represent severe constraints for agriculture; with their combined effects accounting for ~70% of yield losses in legume crops (Huang et al., 2013; Rasool et al., 2013; Rodziewicz et al., 2014; Diaz et al., 2017).

Under natural conditions, both drought and Pi deficiency interfere with plant metabolism via various stress signals and hormonal changes that play essential roles in regulation processes (Ha et al., 2012; Osakabe et al., 2013; López-Arredondo et al., 2014; Cerezini et al., 2016; Srivastava et al., 2016; Nasr Esfahani et al., 2017). Drought and Pi deficiency result in decreases in photosynthetic activity, soluble protein contents, nutrient uptake, and metabolic enzyme activities, causing significant losses in plant growth and biomass, and ultimately in yield components (Garg et al., 2004; Shubhra et al., 2004; Dita et al., 2006; Gobarah et al., 2006; Gunes et al., 2006; Jin et al., 2006; Santos et al., 2006; Kunert et al., 2016; Goufo et al., 2017). Poor nodule development and dis-functioning are frequently detected in legume crops as a result of drought and Pi deficiency, leading to a rapid decrease of nitrogenase activity, and thus BNF efficiency (Sinclair et al., 2007; Charlson et al., 2009; Gil-Quintana et al., 2013; Sulieman et al., 2013; Cabeza et al., 2014; Nasr Esfahani et al., 2014). In many farming areas of the dry savanna of Nigeria, low available soil-Pi levels and poor plant growth are frequent (Nwoke et al., 2003). One of the major reasons of low productivity of legume crops grown in the region is the inefficient BNF resulted from insufficient Pi supply (Sanginga, 2003; Sulieman et al., 2013). Under low available soil-Pi conditions, strategies for improving Pi use are multiple, and include increase of the root surface-soil contact, enhancement of root association with arbuscular mycorrhizal fungi, improvement of rhizosphere modification processes, increases in organic acid exudation, and biotechnological approaches (Hinsinger et al., 2003; Vance et al., 2003; Jones et al., 2004; Ha and Tran, 2014; López-Arredondo et al., 2014; Zhang et al., 2014).

Cowpea (*Vigna unguiculata*) is an important legume crop cultivated by ~10 million smallholder farmers in West Africa. The crop plays an important role in diet, providing diversified sources of proteins and micronutrients for improvement of human nutrition in the West African countries (El-Enany et al., 2013; Mantri et al., 2013). The mean grain yield in sub-Saharan farmers' fields remains very low, less than 500 kg ha^−1^, and far below the achievable potential (Kristjanson et al., 2005; FAOSTAT, 2013). Unfortunately, cowpeas are currently grown on very poor soils under erratic and limiting rainfall conditions, with limited use of fertilizers (e.g., P) in African farming systems (Timko et al., 2007; Abaidoo et al., 2017). Successes in breeding cowpea varieties with greater BNF potential for the various targeted areas largely depend on better understanding of how desirable traits are genetically inherited, as well as the underlying mechanisms, particularly under drought and Pi deficiency. Identification and development of drought-resistant cowpea varieties to benefit resource-poor farmers are the main tasks in breeding activities in Africa (Fatokun et al., 2012; Diaz et al., 2017; Goufo et al., 2017). Several cowpea lines with improved drought resistance at different stages of growth have been identified (Khan et al., 2015; Blum, 2017; Huynh et al., 2017).

Although the combined effects of drought and Pi deficiency are stronger than the effects of each single stress, most of the studies have so far focused on a single stress factor (Fatokun et al., 2012; Abaidoo et al., 2017). Only limited scientific works have investigated the cowpea responses, particularly BNF responses, to the combined effects of these two stresses (Daryanto et al., 2015; Abaidoo et al., 2017). Thus, the objectives of the present work were (i) to assess the effects of soil water conditions (SWC; WW—well-watered and WD—water deficit) to nine Nigerian cowpea varieties with regard to their BNF potential at different levels of available soil-Pi (low, moderate and high available soil-Pi), and (ii) to identify elite cowpea varieties with the best BNF performance under WD and Pi stress conditions in order to assist the cowpea growers to achieve better yields under subsistence farming conditions of the tropics. Our results reported in the present study will provide knowledge that could be applied for improvement of cowpea symbiotic adaptation using biotechnological approaches.

## Materials and methods

### Plant materials

Seeds of nine cowpea varieties were obtained from the legume-breeding unit of the International Insitute of Tropical Agriculture (IITA), Ibadan. The seeds were first surface-sterilized in 95% ethanol for 5 min, washed with sterile distilled water, and subsequently pre-germinated on wet filter papers for five days at 25°C. Growth characteristics and origin of the cowpea varieties are presented in Table S1.

### Experimental soils

An initial field survey was conducted on 41 farmer fields in the Mokwa area (spanning from 9° 08′ to 9° 83′ N and from 5° 21′ to 5° 52′ E) to identify fields with high, moderate, or low levels of available soil-Pi concentrations (Table S2). Sampled fields are all located in the Southern Guinea Savanna agro-ecological zone, and dominant soil types in the area are largely recognized for their low available soil-Pi status. Ten to 15 kg of soil (0–20 cm depth) from each sampled field were collected and brought to the IITA station Ibadan for the bioassay establishment and subsequent laboratory analyses. Cowpea, soybean (*Glycine max*) and groundnut (*Arachis hypogaea*) were the last grown crops before soil sampling.

### Soil analyses

Soil samples were ground and passed through 1-mm mesh to obtain the fine fraction. The pH, and Pi, total N, calcium (Ca), and magnesium (Mg) concentrations in all 41 collected field samples were determined (Table S2). The soil-pH was measured in aqueous soil suspension (1:2.5, v:v) using the Corning 125 pH meter (Corning Life Sciences, Amsterdam, Netherlands) after shaking the samples for 16 h (Table S2). Subsequently, the soil-pH, and soil-Pi, -N, -Ca, and -Mg concentrations of the bulked samples for each soil-Pi level (low, moderate, and high available soil-Pi) were determined (*n* ≥ 8 field samples/soil-Pi level) (Tables S2, S3). The available soil-Pi content was determined using the Bray-I chemical extraction method. Succinctly, 30 mL of Bray-I extractant were added to 3 g air-dried soil. The mixture (1:10, soil:solution ratio) was then shaken for 5 min, filtered, and the inorganic Pi concentration of filtered extract was colorimetrically measured (Murphy and Riley, 1962). For soil-N content analysis, the method described by Novozamsky et al. (1983) was used. For the determination of Ca and Mg concentrations, 30 mL of Mehlich extractant were added to 3 g air-dried soil. The mixture (1:10, soil:solution ratio) was shaken for 5 min and filtered. Ca and Mg concentrations of filtered extract were determined with atomic absorption spectrophotometer (Thermo Scientific iCE 3300 AA Spectrometer, Thermo Scientific, USA).

Data of available Pi levels in soils were used to group fields into three classes of soil-Pi level (Table S2). Out of the 41 farmer fields sampled, 20 fields were very low in available soil-Pi concentration, less than 3 mg Pi kg^−1^ (low available soil-Pi), 13 fields had available soil-Pi concentration between 3 and 10 mg Pi kg^−1^ (moderate soil-Pi), and 8 fields had available soil-Pi concentration above 10 mg Pi kg^−1^ (high available soil-Pi) (Table S2). Soils from the respective classes (20 of low soil-Pi, 13 of moderate-Pi, and 8 of high-Pi) collected from each field as previously described (subsection Experimental Soils) were bulked together and used for the bioassays.

### Bioassays and experimental setup

Approximately 4 kg of fresh soils from different soil-Pi levels were placed into each pot. Four pots for each treatment (*n* = 4) were established, randomly arranged and their positions were re-arranged at weekly intervals under greenhouse conditions. The following factors being tested: (i) factor 1: SWC treatment with 2 levels (WW and WD), (ii) factor 2: soil-Pi availability at three levels: low, moderate and high available soil-Pi, and (iii) factor 3: cowpea varieties with 9 levels (9 different varieties). Five pure seeds of each cowpea variety were sown per pot, and one week after sowing, the cowpea seedlings were thinned to three per pot. Each pot received a modified Jensen's nutrient solution without N and P with the following composition in μM: K_2_SO4, 1,000; CaCl_2_, 360; MgCl_2_, 220; MgSO_4_, 80; MnSO_4_, 4.8; ZnSO_4_, 2; CuSO_4_, 0.8; CoCl_2_, 0.2; H_3_BO_3_ 1.2, (NH_4_)_6_Mo_7_O_4_, 0.2 (Jensen and Collins, 1985). The nutrient solution was applied a week after sowing and at weekly intervals with the daily application of distilled water in between. The pots received water and nutrient solution up to the 4th week after sowing, then watering was stopped for the WD-stressed pots. *In vivo* chlorophyll (Chl) content of plant leaves was measured at weekly intervals following water stress treatments, and was expressed in units of quantity per area of leaf surface (μmol per m^2^ of leaf surface) using a SPAD-502 Plus Chlorophyll Meter (Konica Minolta Optics, Inc., Tokyo, Japan). Karate (Zeon Technology, Syngenta), a foliar-applied residual insecticide was sprayed to plants at 35 and 48^th^ days after sowing (DAS) at concentration of 0.3 mg L^−1^ to prevent occurrence of insects during the course of experiment.

### Water treatment

The amount of water added to pots with stressed plants was measured as follows: the volume of water retained in soil from each pot at field capacity was calculated by determining the wilting point of water content at plant's wilting point subtracted from the field capacity water content. The sample was saturated with water and left to equilibrate overnight. After 24 h the moisture content in the soil sample was left at field capacity. The soil samples were weighed, placed in an oven at 105°C for 2 h and weighed again, and the volume of water to be added was determined as the percentage of added water. Plants were watered every day with deionized water for maintaining 60% water holding capacity in soil until 28 DAS, and then half of the plants were exposed to WD by withholding water supply for another 28 days (a cycle of complete soil dehydration) following the procedure described by Fatokun et al. (2012). After the treatment, samples were harvested from both WW control and WD plants for further analyses. The effect of WD on plant growth, uptake of Pi and N, and BNF potential parameters vs. WW conditions, called “WD reduction” (WDR) effect, on a given parameter, was calculated as below (Equation 1):

(1) $$\begin{array}{l} \text{WDR }(\%)=[(\text{parameter of WW plants-parameter of WD}\\ \text{ plants})\text{/parameter of WW plants}]\;\times \;100 \end{array}$$

In addition, the promoting effect of moderate and high available soil-Pi levels [called “Pi-increasing” (PI) effect] on plant growth, uptake of Pi and N, and BNF potential parameters vs. low available soil-Pi levels was calculated using the following equation (Equation 2):

(2) $$\begin{array}{l} \text{PI }(\%)=[(\text{parameter of plants grown on soil with moderate or}\\ \text{ high available soil-Pi-parameter of plants grown}\\ \text{ on soil with low available soil-Pi})\text{/parameter of}\\ \text{ plants grown on soil with moderate or high available}\\ \text{ soil-Pi}]\;\times \;100 \end{array}$$

### Harvest

At 56th DAS, shoots cut at 5 cm above ground level were collected, and their fresh matter (FM) was recorded. Subsequently, the shoot samples were oven-dried at 70°C for 72 h, and their dry matter (DM) was recorded. Subsamples were placed on water-saturated polyurethane foam in a moist chamber for rehydration, and their turgid matter (TM) was determined. The relative water content (RWC) of plant shoots was determined following the Equation (3).

(3) RWC (%) = [(FM-DM)/(TM−DM)] × 100

Roots were gently washed to remove the soil. The nodules were then removed from roots, and their number was determined, before the FM of nodules and roots were recorded. Lately, fresh roots and nodules were oven-dried at 70°C for 72 h, and their DM was recorded.

### Elemental analyses

Subsamples of shoots and roots were finely ground and passed through 1-mm mesh, and then 0.5 g of each sample was digested in concentrated H_2_SO_4_ at 500°C. The N concentrations in the shoot and root extracts were measured according to the method described by Novozamsky et al. (1983). P contents in shoot and root extracts were determined by the colorimetric procedure of Murphy and Riley (1962).

### Determination of the BNF potential

Sufficient evidence indicates that BNF potential can be accurately assessed by the ureide-N method (Herridge and Peoples, 1990). A highly correlated relationship between the BNF activity and ureide-N content present in the stem extracts has been reported. Thus, the ureide-N method can provide a simple and accurate means for estimating BNF activity. It has been well-known that cowpeas transport fixed-N to shoots as ureides, and reduce nitrate mostly in their shoots. Hence, BNF potential was assessed using the relative ureide-N abundance (RUA) method, based on the quantification of ureide-N and nitrate concentrations in xylem sap (Peoples et al., 1989; Herridge and Peoples, 2002). Shoot sections were taken just above the first node and dried in an oven at 70°C. After removing the leaves, stems and petioles were ground to fine powder to pass through a 40-μm mesh. 0.5 g of stem sample were placed in Pyrex tubes, and were extracted with 15 mL of boiling water for 2–3 min. The mixture was then filtered, made up to 25 mL and frozen for later determination of the ureide-N concentration. The ureide-N concentration was determined following the method of Young and Conway (1942). The nitrate concentration in the solute extract was determined using the salicylic acid method (Cataldo et al., 1975). Absorbances for ureide-N and nitrate concentrations were read at 525 and 410 nm, respectively, using a spectrophotometer (Jenway 6310 Scanning Visible Range Spectrophotometer 230 V, Clarkson Laboratory, USA). Subsequently, the RUA was calculated based on the molar concentration of ureides and nitrate with the assumption of 4 N atoms per ureide molecule, using the following equation below (Jemo et al., 2006):

(4) RUA (%) = 4 N1/(4N2+N1) ×100

where *N1* is the concentration of ureides, and *N2* is the concentration of nitrate in the stem and petiole extracts in nmol.

The BNF potential was then determined following the formula of Herridge et al. (1990) (Equation 5):

(5) $$\begin{array}{l} \text{BNF potential }(\text{mg N }{\text{plant}}^{\text{-1}})\text{ = plant-N}\\ \text{content }(\text{mgN }{\text{plant}}^{\text{-1}})\;\times \text{ RUA} \end{array}$$

In this respect, BNF potential provides estimation for the maximum BNF activity of plants, while N content indicates the actual amount of N fixed by plants that can also be used as an indicator for plant growth.

### Calculation and statistics

Data were statistically analyzed using the “Statistical Analysis System” software version 9.2 (SAS, 2009). Given that the ANOVA common-based approaches can perform multiple comparisons on the main effect means only, a three-way ANOVA model was used to test the variance of each main factor and their interactions on recorded parameters of individual variety. When the Fisher's test denoted a significant effect, the PDIFF option of the least-square means (LSMEANS) was used to perform multiple comparisons between effects of main factors and their interactions for each independent variable. Values in columns followed with the same letter are not significantly different at *p* < 0.05 (LSMEANS/PDIFF option). The principal component (PC) analysis (PCA) of data, transformed by either log (x+1) or square root or arsine square-root, was performed to extract the main differences among varieties under different available soil-Pi levels and SWC.

## Results

### Characteristics of soils

Among 41 soil samples collected from the fields, 20, 13, and 8 samples were identified to belong to low, moderate and high available soil-Pi classes, respectively (Table S2). The mean values ± standard errors of the available soil-Pi concentrations were 1.9 ± 0.11, 5.4 ± 0.6, and 15.3 ± 2.0 mg Pi kg^−1^ for low, moderate and high available soil-Pi level classes, respectively (Table S3). Other chemical properties, such as pH, N, Ca, and Mg concentrations, across the soils of different Pi levels are also presented in Table S3. The soil-pH significantly differed among the three soil groups with higher values recorded in the high available soil-Pi level class. The soil-N, -Ca, and -Mg concentrations were generally correlated with the available soil-Pi levels.

### Plant Chl content and RWC

The effects of SWC, available soil-Pi levels and cowpea varieties were highly significant (*p* < 0.001) with regard to the Chl content (Table 1). Plants under WW conditions displayed greater Chl contents than under WD conditions (Table 2). The cowpea varieties exhibited variations from each other in terms of Chl contents under WD and available low soil-Pi conditions (Table 2). Similarly, under moderate available soil-Pi supply, significant variations between the WD and WW plants were noted, and the majority of plants under WW conditions displayed at least two-fold, dependently on the variety, higher in Chl content than under WD conditions (Table 2). With high available soil-Pi supply, varieties (except IT04K-405-5) grown under WW conditions produced significantly higher Chl contents compared with those under WD (Table 2). With regard to the interaction effects tested among SWC, cowpea varieties (V) and available soil-Pi (Pi) levels, we observed that the SWC × V interaction displayed significant (*p* < 0.05) effects on Chl content, while other types of interactions did not (Table 1).

**Table 1:** Results of the three-way ANOVA testing the effects of soil water conditions (SWC; two levels; WW, well-watered; WD, water deficit), available soil-phosphate (Pi) levels (three levels; low available soil-Pi, moderate available soil-Pi and high available soil-Pi), cowpea varieties (V, nine varieties), and their interactions on chlorophyll (Chl) content, shoot relative water content (RWC), nodule number, biomass production, biological nitrogen fixation (BNF) potential, plant-N and -P contents of cowpea varieties.

	**SWC**	**Available soil-Pi**	**V**	**V × Pi**	**SWC × V**	**SWC × Pi**	**SWC × Pi × V**
Chl content [μmol per m^2^ of leaf surface]	854.0 (^***^)	123.6 (^***^)	187.2 (^***^)	6.0 (ns)	104 (^*^)	5.8 (ns)	40 (ns)
Shoot RWC [%]	6203 (^***^)	1.1 (ns)	0.5 (ns)	0.3 (ns)	0.8 (ns)	1.7 (ns)	0.3 (ns)
Nodule number (plant^−1^)	670.5 (^***^)	78.8 (^***^)	19.9 (^***^)	3.2 (^*^)	20.1 (^***^)	76.3 (^***^)	3.6 (^*^)
Shoot-to-root ratio [(g DM shoot)/(g DM root)]	179.5 (^***^)	4.5 (^*^)	1.4 (ns)	2.1 (ns)	3.3 (^**^)	2.0 (ns)	0.3 (ns)
Plant (shoot + root) biomass DM [g plant^−1^]	519.4 (^***^)	24.1 (^***^)	1.3 (ns)	0.6 (ns)	1.4 (ns)	9.5 (^***^)	1.0 (ns)
Plant (shoot + root)-P content [mg P plant^−1^]	519.4 (^***^)	33.6 (^***^)	2.0 (ns)	0.4 (ns)	1.3 (ns)	19.9 (^***^)	0.3 (ns)
Plant (shoot + root)-N content [mg N plant^−1^]	741.2 (^***^)	80.2 (^***^)	4.3 (^**^)	0.7 (ns)	3.0 (^*^)	37.8 (^***^)	1.8 (ns)
BNF potential [mg N plant^−1^]	341.8 (^***^)	41.3 (^***^)	3.8 (^*^)	0.7 (ns)	2.0 (^*^)	19.9 (^***^)	0.7 (ns)

The F-values along with the level of significance are presented. Four replicates per treatment were tested. Asterisks indicate significant differences as determined by Fisher's test (

* p < 0.05;

** p < 0.01;

*** *p < 0.001; ns, not significant). DM, dry matter*.

**Table 2:** Chlorophyll (Chl) content and shoot relative water content (RWC) of cowpea varieties under different available soil-phosphate (Pi) levels (low available soil-Pi, moderate available soil-Pi, and high available soil-Pi) and soil water conditions (WW, well-watered; WD, water deficit).

**Variety**	**Low soil-Pi**		**Moderate soil-Pi**		**High soil-Pi**	
	**WD**	**WW**	**WD**	**WW**	**WD**	**WW**
**CHL CONTENT (**μ**mol per m**^2^ **OF LEAF SURFACE)**						
Dan'Ila	30.8 ± 2.5 Ab	53.8 ± 0.2 Ca	27.9 ± 0.5 Bb	52.0 ± 2.0 Ca	25.1 ± 0.9 ABb	61.4 ± 0.6 Aa
IT04K-339-1	18.8 ± 2.3 CDb	52.5 ± 2.6 Ca	22.1 ± 0.5 Db	52.2 ± 2.2 Ca	27.5 ± 4.0 ABb	58.4 ± 2.1 BCa
IT04K-405-5	21.7 ± 2.3 Cb	52.7 ± 2.5 Ca	23.9 ± 1.6 Cb	50.8 ± 2.6 Ca	22.7 ± 1.0 Ba	47.6 ± 1.0 Da
IT06K-281-1	27.2 ± 1.5 ABb	49.0 ± 4.8 CDa	26.7 ± 3.6 BCb	55.9 ± 1.1 Ba	21.7 ± 3.1 Bb	57.3 ± 0.7 Ba
IT07K-187-24	21.0 ± 3.1 Cb	54.8 ± 1.5 BCa	33.6 ± 2.3 Ab	63.6 ± 0.8 Aa	28.8 ± 3.6 Ab	54.2 ± 2.1 Ca
IT07K-188-49	19.5 ± 2.1 CDb	56.1 ± 0.2 Ba	21.7 ± 3.9 CDb	62.8 ± 3.9 Aa	21.1 ± 5.8 ABb	58.6 ± 2.1 BCa
IT07K-304-9	19.6 ± 3.9 CDb	47.5 ± 1.5 Da	24.9 ± 1.7 Cb	50.5 ± 2.2 Ca	26.7 ± 3.2 ABb	54.4 ± 1.9 Ca
IT97K-499-35	14.9 ± 2.6 Db	59.0 ± 2.0 Aa	32.2 ± 4.5 ABb	61.9 ± 2.7 Aa	24.5 ± 2.1 ABb	63.7 ± 2.5 Aa
TVU7778	26.2 ± 1.5 Bb	42.8 ± 1.2 Ea	15.0 ± 0.5 Eb	46.5 ± 0.9 Da	11.5 ± 1.1 Cb	42.9 ± 1.0 Ea
**SHOOT RWC (%)**						
Dan'Ila	64.8 ± 2.5 Ab	80.7 ± 0.6 Ca	82.0 ± 3.0 Ab	84.2 ± 0.6 Aa	82.6 ± 2.7 Ab	83.6 ± 0.7 Ba
IT04K-339-1	63.5 ± 2.3 Ab	80.9 ± 0.6 Ca	72.4 ± 3.2 Bb	84.4 ± 1.5 Aa	74.6 ± 2.8 Cb	82.5 ± 0.5 Ba
IT04K-405-5	56.3 ± 1.2 Bb	87.8 ± 0.3 Aa	74.6 ± 4.6 Ba	84.5 ± 3.4 Aa	56.6 ± 2.8 Ea	85.4 ± 0.3 Aa
IT06K-281-1	46.6 ± 1.5 Cb	83.8 ± 0.4 Ba	71.2 ± 3.7 Bb	82.8 ± 0.6 Aa	71.9 ± 4.5 CDb	81.9 ± 0.7 Ba
IT07K-187-24	62.3 ± 1.5 Ab	78.7 ± 0.4 Da	64.8 ± 2.9 Cb	82.4 ± 1.6 Aa	75.2 ± 3.4 BCb	79.3 ± 0.9 Da
IT07K-188-49	60.6 ± 3.4 Bb	73.8 ± 0.8 Ea	79.3 ± 1.6 Ab	80.1 ± 0.5 Ba	81.9 ± 4.8ABb	79.3 ± 1.0 Da
IT07K-304-9	69.0 ± 3.2 Ab	84.3 ± 0.8 Ba	73.3 ± 2.5 Bb	83.7 ± 0.3 Aa	72.6 ± 4.0 CDb	81.0 ± 0.5 Ca
IT97K-499-35	64.9 ± 4.3 Ab	75.3 ± 1.1 Da	74.8 ± 2.6 Bb	78.8 ± 0.9 Ca	66.3 ± 3.8 Da	81.3 ± 2.0 BCa
TVU7778	67.5 ± 4.7 Ab	86.3 ± 1.2 Aa	54.4 ± 1.9 Db	84.5 ± 0.6 Aa	65.0 ± 3.3 Db	82.1 ± 1.0 Ba

*Mean values and standard errors are shown (n = 4). Different small letters indicate significant differences between the WD and WW plants for each variety at each available soil-Pi level (Fisher's test; p < 0.05). Different capital letters indicate significant differences among the varieties at each available soil-Pi level and WD conditions (Fisher's test; p < 0.05)*.

The RWCs of plant shoots analyzed at 56th DAS exhibited highly significant (*p* < 0.001) differences following the imposed WD stress (Table 1). Plants under WW conditions displayed the highest values of RWC with various levels of available soil-Pi supply (Table 2). The RWC ranged from 46.6 to 87.8%, with IT06K-281-1 variety displaying the lowest RWC (46.6%) under WD and low available soil-Pi, while IT04K-405-5 variety exhibiting the highest RWC (87.8%) under WW and low available soil-Pi conditions (Table 2). Importantly, plants under WD conditions and high available-Pi supply maintained the highest RWC compared with that under WD and low-available-Pi conditions (Table 2).

### Cowpea DM

**Figure 1:**
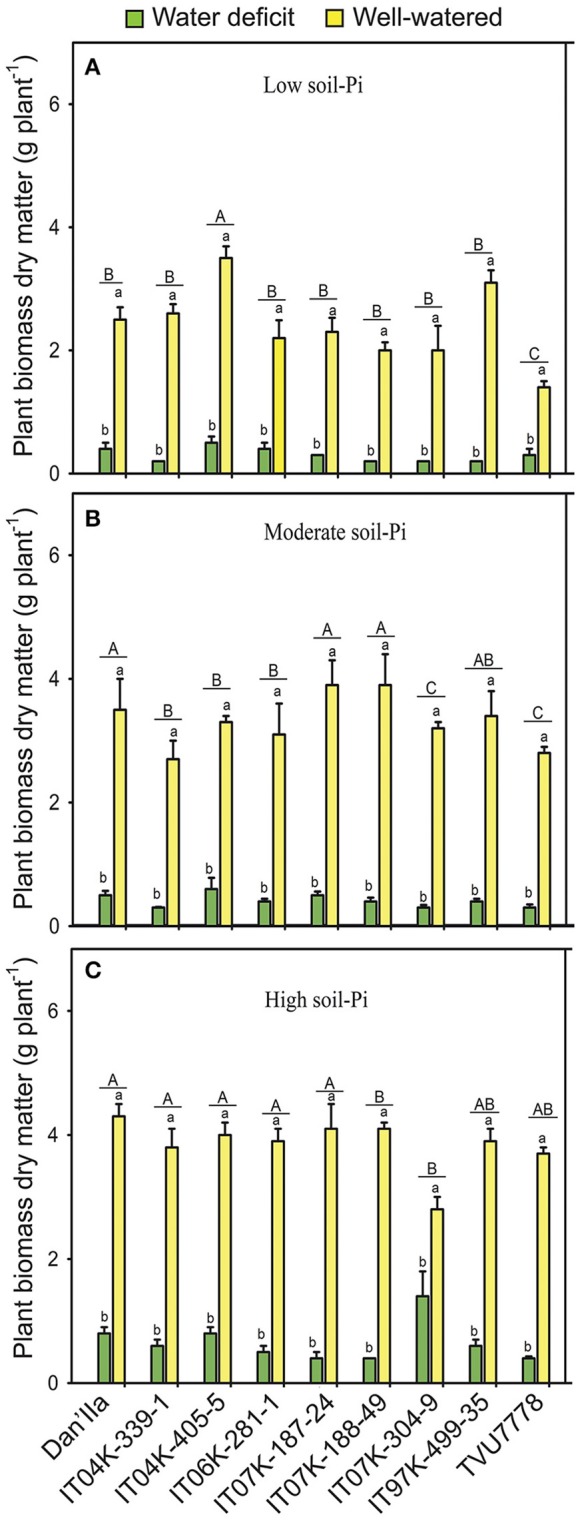
Plant biomass dry matters of cowpea varieties under different available soil-phosphate (Pi) levels with or without water deficit stress. **(A)** Low soil-Pi, **(B)** moderate soil-Pi, and **(C)** high soil-Pi. Mean values and standard errors (bars) are shown (*n* = 4). Different small letters indicate significant differences between the water-deficit and well-watered plants for each variety at each available soil-Pi level (Fisher's test; *p* < 0.05). Different capital letters indicate significant differences among the varieties at each available soil-Pi level (Fisher's test; *p* < 0.05).

We observed significant DM differences between the WD and WW plants under low available soil-Pi levels, with plants on sufficient water exhibiting higher DM compared with the WD ones (Figure 1A). All the cowpea varieties tested produced remarkably higher DM on WW than WD conditions under any available soil-Pi levels (Figures 1A–C). Plant DM reductions by WD ranged from 65.1 to 92.3%, with IT04K-339-1 showing the lowest (65.1%) under high available soil-Pi and IT97K-499-35 the highest (92.3%) plant biomass reduction under moderate available soil-Pi, respectively, after 28 days of WD treatment (Figure S1A).

Several cowpea varieties, such as IT04K-339-1 and IT04K-405-5, exhibited remarkable different DMs under WD and low available soil-Pi conditions, or under WD and moderate available soil-Pi supply (Figures 1A,B). Under WW and low available soil-Pi conditions, cowpea varieties recorded differences in DM, with varieties IT04K-405-5 and IT97K-499-35 produced higher DM than others (Figure 1A). The cowpea varieties grown under WW conditions with moderate available soil-Pi supply displayed different DM accumulation (Figure 1B). For instance, varieties Dan'Ila, IT07K-187-24 and IT07K-188-49 accumulated higher DM than other varieties (Figure 1B). Under WW and high available soil-Pi supply conditions, there were no large variations in DM among the tested cowpea varieties, with exception of IT07K-304-9 that surprisingly showed the lowest DM (Figure 1C).

The ANOVA testing the effects of available soil-Pi levels on DM of cowpea varieties revealed highly significant (*p* < 0.001) differences among the available soil-Pi levels (Table 1). The plant DM increases due to soil-Pi availabilities ranged from −7 to 69.4% with varieties IT04K-405-5 and IT07K-304-9 showing the lowest (−7%) and the highest DM increases (69.4%) under high and moderate available soil-Pi levels, respectively, when compared with low available soil-Pi level (Figure S2A). Under both WD and WW conditions, high available soil-Pi level had higher increasing effect on plant DM, with exception of that of IT04K-405-5, in comparison with low and moderate available soil-Pi levels (Figure S2A). Under WD, moderate soil-Pi level improved better the DM of IT07K-188-49 and IT97K-499-35 than that of other varieties (Figure S2A). Under WW conditions, moderate available Pi level also showed more positive effect on DM of the cowpea varieties than low-Pi supply, with variety IT07K-304-9 displaying the highest increase in plant biomass DM (Figure S2A). In addition, the SWC × Pi interaction was highly significant at *p* < 0.001 (Table 1). At high, moderate and low available soil-Pi supplies, all the cowpea varieties showed higher DM, by at least two-fold (except IT07K-188-49), dependently on the variety, under WW conditions in comparison with WD (Figure S2A). With sufficient water supply, the majority of the cowpea varieties grown on soil with high available soil-Pi level gained higher DM than those on soil with moderate or low available soil-Pi level (Figure 1).

### Shoot/root ratio

The ANOVA indicated that the effects of SWC on shoot/root ratio were highly significant at *p* < 0.001 (Table 1). Under WD and low available soil-Pi level, the cowpea varieties displayed differences in shoot/root ratio, with variety Dan'Ila exhibiting the highest value (Table 3). Under low available soil-Pi and WW conditions, there were no remarkable differences in the shoot/root ratio among the cowpea varieties, although variety Dan'Ila recorded the highest shoot/root value (Table 3). When grown under moderate available soil-Pi level and WD conditions, the cowpea varieties tended to lower their shoot/root values, except varieties Dan'Ila and TVU7778, but differences were still noticed among the varieties (Table 3). With moderate available soil-Pi supply, plants grown on WW conditions displayed variations in shoot/root ratio, and almost all the varieties, except Dan'Ila, showed shoot/ratio values of > two-fold than that under WD (Table 3). In addition, we also observed variations in shoot/root ratio of the cowpea varieties under WD and high available soil-Pi supply, and varieties IT04K-405-5 and IT07K-304-9 displayed the highest shoot/root ratio (Table 3). When subjected to WW and high available soil-Pi supply, there were differences in shoot/root ratio among the cowpea varieties, with IT07K-304-9 and IT97K-499-35 exhibiting the greatest shoot/root values (Table 3).

**Table 3:** Shoot/root ratio, nodule number and plant-nitrogen (N) content of cowpea varieties under different available soil-phosphate (Pi) levels (low available soil-Pi, moderate available soil-Pi, and high available soil-Pi) and soil water conditions (WW, well-watered; WD, water deficit).

	**Low soil-Pi**		**Moderate soil-Pi**		**High soil-Pi**	
	**WD**	**WW**	**WD**	**WW**	**WD**	**WW**
**SHOOT/ROOT RATIO [(g DM shoot)/(g DM root)]**						
Dan'Ila	4.4 ± 0.4 Aa	5.6 ± 0.9 Aa	3.8 ± 0.6 Aa	5.1 ± 0.1 Ba	1.7 ± 0.3 Bcb	5.6 ± 0.6 Ca
IT04K-339-1	1.3 ± 0.3 Db	4.7 ± 0.7 Aa	1.7 ± 0.2 CDb	4.3 ± 0.3 Ca	2.5 ± 0.1 Bb	6.1 ± 0.3 Ca
IT04K-405-5	2.1 ± 0.2 Cb	4.5 ± 0.8 Aa	1.5 ± 0.1 CDb	4.1 ± 0.4 Ca	5.9 ± 1.2 Aa	5.0 ± 0.7 Ca
IT06K-281-1	3.2 ± 0.4 Bb	5.0 ± 0.8 Aa	1.5 ± 0.5 CDb	5.0 ± 0.3 Ba	2.6 ± 0.8 Bb	5.8 ± 0.6 Ca
IT07K-187-24	1.6 ± 0.4 Cb	3.3 ± 0.2 Ba	2.3 ± 0.8 Bcb	6.4 ± 1.1 ABa	1.5 ± 0.8 Bcb	5.3 ± 0.6 Ca
IT07K-188-49	2.0 ± 0.4 Cb	5.5 ± 0.9 Aa	1.9 ± 0.2 Cb	7.8 ± 1.0 Aa	1.5 ± 0.2 Cb	6.3 ± 1.0 Bca
IT07K-304-9	1.0 ± 0.2 Db	4.6 ± 0.8 Aa	1.9 ± 0.1 Cb	6.5 ± 1.2 ABa	4.4 ± 0.4 Ab	7.9 ± 0.9 ABa
IT97K-499-35	2.7 ± 0.5 Bcb	4.8 ± 0.3 Aa	1.3 ± 0.3 Db	7.6 ± 1.2 ABa	2.4 ± 0.8 Bcb	8.7 ± 0.5 Aa
TVU7778	2.3 ± 0.1 Cb	5.3 ± 0.1 Aa	2.7 ± 0.2 Ba	5.7 ± 0.7 Ba	2.1 ± 0.3 Bb	5.0 ± 0.7 Ca
**NODULE NUMBER [plant**^−1^**]**						
Dan'Ila	0.0 ± 0.0 Bb	17.2 ± 2.5 Ba	0.0 ± 0.0 Bb	31.2 ± 3.9 Ba	0.0 ± 0.0 Bb	62.5 ± 7.3 Aa
IT04K-339-1	0.0 ± 0.0 Bb	12.3 ± 1.6 Ca	0.0 ± 0.0 Bb	14.0 ± 3.6 Da	3.2 ± 0.3 Ab	36.7 ± 2.7 Ca
IT04K-405-5	0.8 ± 0.1 Ab	22.3 ± 1.6 Aa	0.0 ± 0.0 Bb	57.3 ± 5.7 Aa	0.0 ± 0.0 Bb	69.2 ± 5.1 Aa
IT06K-281-1	0.0 ± 0.0 Bb	7.2 ± 0.1 Da	0.0 ± 0.0 Bb	15.5 ± 4.5*C* Da	0.0 ± 0.0 Bb	43.5 ± 1.8 Ba
IT07K-187-24	0.0 ± 0.0 Bb	11.3 ± 2.3 Ca	2.2 ± 0.2 Ab	12.3 ± 2.2 Da	0.0 ± 0.0 Bb	29.0 ± 2.0 Da
IT07K-188-49	0.0 ± 0.0 Bb	5.3 ± 0.6 Da	0.0 ± 0.0 Bb	12.0 ± 3.5 Da	0.0 ± 0.0 Bb	11.3 ± 1.4 Ea
IT07K-304-9	0.0 ± 0.0 Bb	15.2 ± 1.3 Bca	0.0 ± 0.0 Bb	29.7 ± 3.2 Ba	0.0 ± 0.0 Bb	67.0 ± 5.5 Aa
IT97K-499-35	0.0 ± 0.0 Bb	6.0 ± 1.9 Da	0.0 ± 0.0 Bb	9.8 ± 1.3 Da	0.0 ± 0.0 Bb	24.7 ± 2.3 Da
TVU7778	0.0 ± 0.0 Bb	17.7 ± 2.2 Ba	0.0 ± 0.0 Bb	21.3 ± 2.1 Ca	0.0 ± 0.0 Bb	38.0 ± 3.1 Bca
**PLANT-N CONTENT [mg N plant**^−1^**]**						
Dan'Ila	8.0 ± 0.9*A* Bb	47.5 ± 2.3 Aa	14.7 ± 1.4 Bb	78.7 ± 5.2 Aa	13.2 ± 1.2 Cb	100.2 ± 7.1 ABa
IT04K-339-1	6.2 ± 1.1 Bcb	40.7 ± 1.9 Ba	9.0 ± 0.2 Cb	52.8 ± 4.7 Ca	12.8 ± 0.3 Cb	77.9 ± 2.7 Ca
IT04K-405-5	9.1 ± 0.2 Ab	52.5 ± 2.9 Aa	16.7 ± 0.3 Ab	70.0 ± 5.6 Aa	23.9 ± 0.3 Bb	100.3 ± 4.7 Aa
IT06K-281-1	6.8 ± 1.1 Bcb	29.5 ± 1.1 Da	10.8 ± 0.5 Cb	48.0 ± 1.4 Da	13.3 ± 1.2 Cb	86.4 ± 7.7 Bca
IT07K-187-24	6.9 ± 0.3 Bcb	40.3 ± 2.7 Ba	9.3 ± 0.7 Cb	67.8 ± 3.9 Ba	11.5 ± 1.3 Cb	84.8 ± 5.1 Ba
IT07K-188-49	4.6 ± 0.8 Db	10.6 ± 2.8 Ea	5.8 ± 0.9 Db	65.4 ± 3.5 Ba	11.4 ± 1.2 Cb	72.3 ± 3.5*C* Da
IT07K-304-9	5.9 ± 0.9 CDb	37.4 ± 3.2 Bca	10.6 ± 1.1 Cb	51.5 ± 3.1 Da	26.6 ± 1.9 Ab	62.2 ± 1.3 Ea
IT97K-499-35	7.0 ± 0.5 Bcb	33.8 ± 1.6 Ca	9.4 ± 0.2 Cb	47.8 ± 2.5 Da	13.9 ± 0.6 Cb	69.4 ± 1.0 Da
TVU7778	7.1 ± 1.4 Bcb	29.4 ± 1.7 Da	9.8 ± 0.4 Cb	59.5 ± 4.8 Bca	13.1 ± 1.0 Cb	86.2 ± 2.1 Ba

*Mean values and standard errors are shown (n = 4). Different small letters indicate significant differences between the WD and WW plants for each variety at each available soil-Pi level (Fisher's test; p < 0.05). Different capital letters indicate significant differences among the varieties at each available soil-Pi level and WD conditions (Fisher's test; p < 0.05). DM, dry matter*.

At various Pi levels and water conditions, the shoot/root ratio of the cowpea varieties ranged from 1.0 to 8.7, with variety IT07K-304-9 showing the lowest shoot/root ratio at low soil-Pi level under WD, while variety IT97K-499-35 displaying the highest value under WW and high available soil-Pi conditions (Table 3). In addition, the effect of SWC × V interaction was found to be highly significant (*p* < 0.01) on the shoot/root ratio (Table 1), indicating that a number of cowpea varieties adjusted their shoot/root ratio under WD for better adaptation (Table 3). The V × Pi, SWC × Pi, and SWC × Pi × V interactions revealed no significant effects on the shoot/root ratio of the cowpea varieties (Table 1).

### Cowpea nodulation

Damage imposed by WD was greatly severe, affecting the nodulation of all nine cowpea varieties, irrespective of the available soil-Pi supply, thereby resulting in almost no nodules in the majority of the varieties, with the exception of IT04K-405-5 (under low available soil-Pi), IT07K-187-24 (under moderate available soil-Pi) and IT04K-339-1 (under high available soil-Pi) (Table 3). When grown under moderate available soil-Pi level, the WW cowpea varieties produced more nodules than the WD ones, with genetic variation being observed, and variety IT04K-405-5 displaying the highest number of nodules (Table 3). Under high available soil-Pi level and WW conditions, all the cowpea varieties produced visible nodules, with the nodule number ranging from 11.3 to 69.2 per plant (Table 3). In addition, except variety IT07K-188-49 all other varieties grown under high available soil-Pi possessed nodule number higher than those grown under low or moderate available soil-Pi supply (Table 3). Furthermore, we observed that the V × Pi, SWC × V, SWC × Pi, and SWC × V × Pi interactions had significant effects on the nodule number per plant (Table 1).

### P content of the cowpea varieties

**Figure 2:**
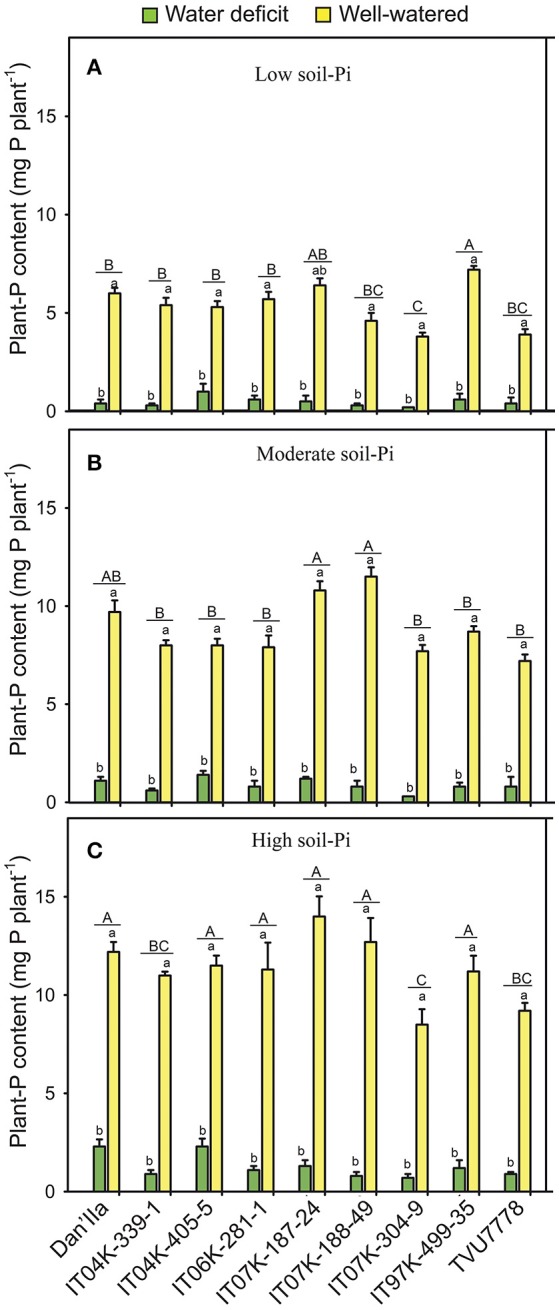
Plant-phosphorus (P) contents in cowpea varieties under different available soil-phosphate (Pi) levels with or without water deficit stress. **(A)** Low soil-Pi, **(B)** moderate soil-Pi, and **(C)** high soil-Pi. Mean values and standard errors (bars) are shown (*n* = 4). Different small letters indicate significant differences between the water-deficit and well-watered plants for each variety at each available soil-Pi level (Fisher's test; *p* < 0.05). Different capital letters indicate significant differences among the varieties at each available soil-Pi level (Fisher's test; *p* < 0.05).

Highly significant (*p* < 0.001) differences in plant-P content between WD and WW cowpea plants were observed (Table 1). Under WD and low available soil-Pi, the cowpea varieties exhibited variations in plant-P content, with variety IT04K-405-5 displaying the highest value (Figure 2A). In addition, during WD and with moderate available soil-Pi supply, varieties Dan'Ila, IT04K-405-5 and IT07K-187-24 showed the highest plant-P contents (Figure 2B), whereas under WD and high available soil-Pi, Dan'Ila and IT04K-405-5 recorded the greatest plant-P contents (Figure 2C). Similarly, genotypic variations in plant-P content were observed under WW conditions and at any soil-Pi supplies (Figure 2). Specifically, with sufficient water availability varieties IT07K-187-24 and IT97K-499-35 recorded the highest plant-P contents at low available soil-Pi supply (Figure 2A), while varieties IT07K-188-49 and IT07K-187-24 produced the greatest plant-P values at moderate soil-Pi availability (Figure 2B). Furthermore, Dan'Ila, IT07K-187-24 and IT07K-188-49 displayed the highest plant-P contents under WW and high soil-Pi availability (Figure 2C). In addition, WD caused a severe reduction in plant-P content, ranging from 63.4 to 95.0% relative to sufficient water supply, with variety IT07K-304-9 showing the lowest plant-P content reduction (63.4%) under high soil-Pi availability, and the same variety (IT07K-304-9) displaying the highest plant-P content reduction (95.0%) under low available soil-Pi conditions (Figure S1B).

The effect of soil-Pi availability on increases of plant-P content ranged from 16.4 to 80.7% vs. that of respective values obtained from low soil-Pi availability, with variety IT97K-499-35 of the high available soil-Pi and WD conditions displaying the lowest increase, while variety Dan'Ila of the moderate available soil-Pi and WW conditions recording the highest increase in plant-P content (Figure S2B). We noticed that cowpea varieties under WW conditions resulted in relatively higher increases in plant-P content than those under WD (Figure S2B). Variety Dan'Ila recorded superior increase in plant-P content in comparison with other varieties under WD and moderate available soil-Pi supply (Figure S2B). Under sufficient water supply, variety Dan'Ila and IT07K-188-49 exhibited the highest increase in plant-P content for the moderate and high soil-Pi levels, respectively (Figure S2B). Among the interactions tested, only the SWC × Pi interaction revealed highly significant (*p* < 0.001) differences in terms of plant-P content among various cowpea varieties (Table 1).

### N content in cowpea varieties

Plants grown under WW conditions displayed significantly higher plant-N contents than those under WD conditions, irrespective of the available soil-Pi supply (Table 3). Under low available soil-Pi and WD conditions, variations in plant-N content were observed among the cowpea varieties, with Dan'Ila and IT04K-405-5 recording the highest plant-N contents (Table 3). Cowpea varieties grown under low available soil-Pi and WW conditions also showed variations, and Dan'Ila and IT04K-405-5 recorded the greatest plant-N contents (Table 3). With respect to moderate available soil-Pi supply and WD conditions, the cowpea varieties exhibited differences from each other, revealing the highest plant-N contents in varieties Dan'Ila and IT04K-405-5 (Table 3). At moderate soil-Pi availability and sufficient water supply, the cowpea varieties differed from each other in plant-N level, with varieties Dan'Ila, IT04K-405-5 and IT07K-187-24 exhibiting the highest plant-N contents (Table 3). Similarly, plants under high available soil-Pi supply and WD conditions were different in plant-N level, with IT04K-405-5 and IT07K-304-9 producing the greatest plant-N contents (Table 3). When investigating the WD effects on plant-N content, plant-N content reductions were ranged from 47.1 to 91.1%, with variety IT07K-188-49 displaying the highest reduction under low available soil-Pi, while the same variety recording the lowest reduction under high available soil-Pi supply (Figure S1C). Importantly, when the cowpea varieties exposed to WD and supplied with high available soil-Pi, the reduction effect of WD was always lowered (Figure S1C).

The effect of soil-Pi availability on cowpea varieties was highly significant (*p* < 0.001) (Table 1), showing significantly greater plant-N content under high available soil-Pi than low or moderate available soil-Pi supply (Table 3). Furthermore, the effects of moderate and high available soil-Pi levels significantly differed in plant-N content of the tested cowpea varieties, irrespective of the SWC (Figure S2C). Obviously, plants under WW and high available soil-Pi conditions exhibited greater increase in plant-N content as compared with those under WD and moderate, or WD and low available soil-Pi conditions (Figure S2C). With respect to the interactions among SWC, V, and Pi, we observed that the SWC × Pi and SWC × V interactions showed significant effects on plant-N content, whereas V × Pi and SWC × V × Pi interactions did not (Table 1).

### BNF potential of cowpea plants

**Figure 3:**
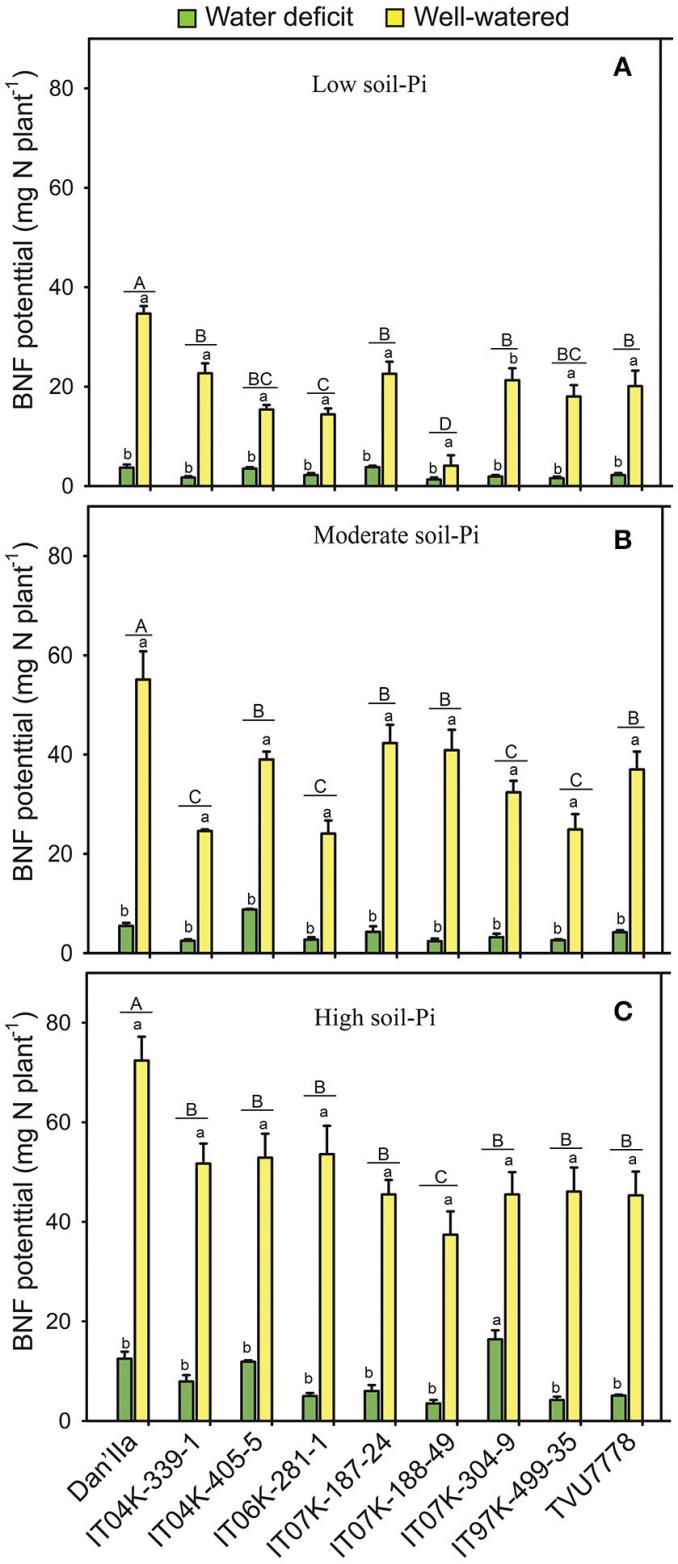
Biological nitrogen fixation (BNF) potential in cowpea varieties under different available soil-phosphate (Pi) levels with or without water deficit stress. **(A)** Low soil-Pi, **(B)** moderate soil-Pi, and **(C)** high soil-Pi. Mean values and standard errors (bars) are shown (*n* = 4). Different small letters indicate significant differences between the water-deficit and well-watered plants for each variety at each available soil-Pi level (Fisher's test; *p* < 0.05). Different capital letters indicate significant differences among the varieties at each available soil-Pi level (Fisher's test; *p* < 0.05).

In general, lower BNF potentials were observed at any available soil-Pi levels under WD than WW conditions (Figure 3). The effects of WD on the BNF potential reduction ranged from 57.1 to 94.6% for the plants grown under low, moderate and high available soil-Pi supplies (Figure S1D). Under low available soil-Pi and WD conditions, we recorded variations among the cowpea varieties, and Dan'Ila, IT04K-405-5 and IT07K-187-24 recorded the highest BNF potential (Figure 3A). Differences in BNF potential of the cowpea varieties grown under WW and low soil-Pi conditions were also observed, with variety Dan'Ila exhibiting the highest BNF potential (Figure 3A). On the contrary, there were no differences among the varieties in terms of BNF potential under WD and moderate available soil-Pi conditions (Figure 3B). Under WW and moderate available soil-Pi conditions, variations in BNF potential were observed among the cowpea varieties, with Dan'Ila displaying the highest BNF potential (Figure 3B). Under WD and high soil-Pi availability, differences in BNF potential of the cowpea varieties were noted, with IT07K-304-9 showing the highest BNF potential (Figure 3C). Similarly, variations in BNF potential of the cowpea varieties were observed under WW and high available soil-Pi conditions, with variety Dan'Ila exhibiting the highest BNF potential (Figure 3C).

The effects of available soil-Pi levels on the BNF potential were highly significant (*p* < 0.001; Table 1). Specifically, the PI effects were at least 20% higher for all tested cowpea varieties, except IT04K-339-1 (under high available soil-Pi and WD), compared with low available soil-Pi under WD conditions (Figure S2D). Similarly, the effects of soil-Pi availabilities on increases in BNF potential were revealed for the cowpea varieties under WD and WW conditions, when comparing the differences between moderate and high available soil-Pi levels against the low available soil-Pi (Figure S2D). Under WW conditions, IT07K-188-49 and IT04K-187-24 recorded the highest and lowest increase in BNF potential with high and moderate available soil-Pi supply, respectively (Figure S2D). Concerning the interaction effects on the BNF potential of the cowpea varieties, the SWC × V and SWC × Pi interactions were significant (*p* < 0.05) and highly significant (*p* < 0.001), respectively (Table 1). However, the interactions V × Pi and SWC × Pi × V resulted in no significant effects on the cowpea BNF potential (Table 1).

### Identification of cowpea varieties with improved performance under WD and low soil-Pi conditions

**Figure 4:**
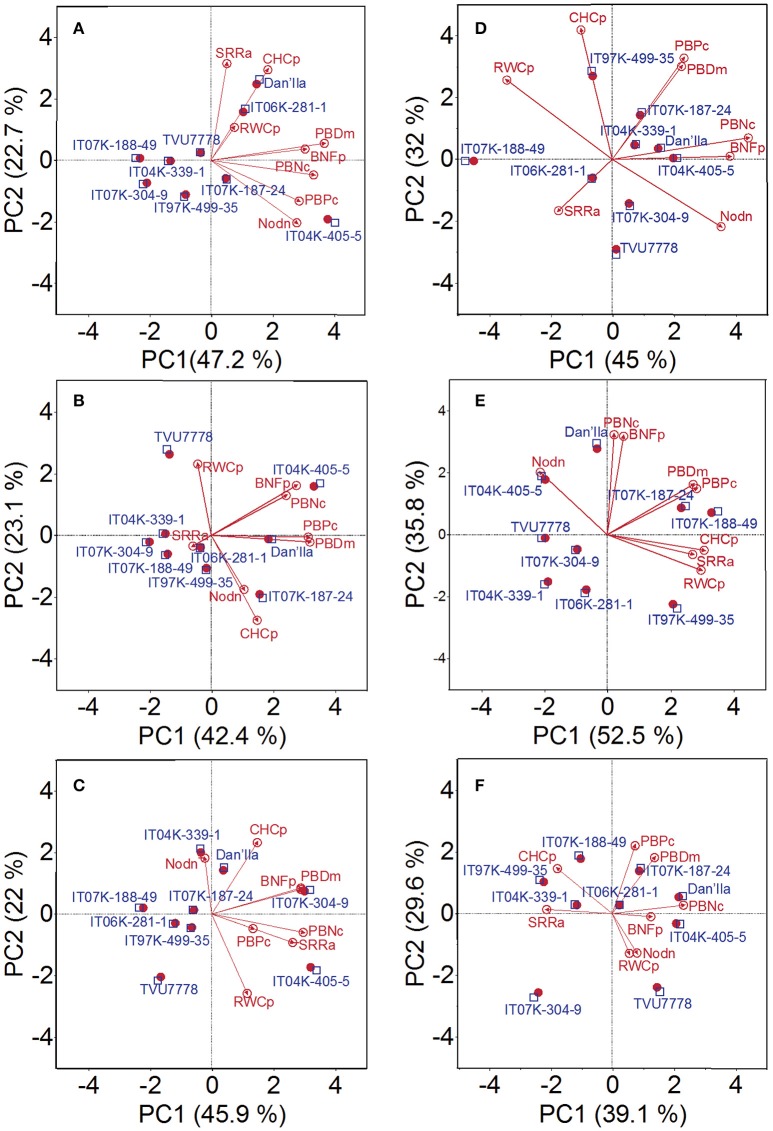
Principal component (PC) analysis displaying the cowpea varieties under **(A)** water deficit and low available soil-Pi level, **(B)** water deficit and moderate available soil-Pi level, **(C)** water deficit and high available soil-Pi, **(D)** well-watered and low available soil-Pi, **(E)** well-watered and moderate available soil-Pi, and **(F)** well-watered and high available soil-Pi conditions. PCs 1 and 2, and their respective contributions are indicated. Nodn, nodule number; SRRa, shoot/root ratio; PBDm, plant (shoot + root) biomass dry matter; PBPc, plant (shoot + root) P content; PBNc, plant (shoot + root) N content; BNFp, biological nitrogen fixation potential; RWCp, relative water content of plant shoots; CHCp, chlorophyll content.

Under WD and available low soil-Pi conditions, the PCs 1, 2, and 3 explained 47.2, 22.7, and 16.8% variability of the data, respectively (Figure 4A; Table S4). Plant parameters displaying the highest positive loading value in the direction of PC1 were nodule number (0.38), plant DM (0.50), plant-P content (0.39), plant-N content (0.45), and BNF potential (0.42) (Table S4). Cowpea varieties in the direction of PC1 were varieties IT04K-339-1, IT07K-188-49, IT07K-304-9, and IT04K-405-5 (Figure 4A). On the other hand, shoot/root ratio (0.61) and Chl content (0.57) exhibited the highest positive loading score for PC2, and show correlation with variety Dan'Ila (Figure 4A; Table S4). Under WD and moderate available soil-Pi conditions, the PC1, PC2, and PC3 explained 42.4, 23.1, and 16.4% of data variability, respectively (Figure 4B; Table S4). With respect to PC1, variables with the highest positive loading score were plant DM (0.52), plant-P content (0.51), plant-N content (0.40), and BNF potential (0.45), while plant RWC (0.51) displayed the highest positive loading score with regard to PC2 (Table S4). In addition, PCs of variables under WD and high available soil-Pi conditions indicated that PC1, PC2, and PC3 could interpret 45.9, 22, and 12.9% of data variability, respectively (Figure 4C; Table S4). Variables shoot/root ratio (0.43), plant DM (0.47), plant-N content (0.48), and BNF potential (0.47) were found to have the highest positive scores for PC1; while Chl content and RWC showed the highest positive and the lowest negative score, respectively, for PC2 (Table S4).

With respect to cowpea varieties grown under WW and low available soil-Pi conditions, PC1 explained higher data variability (45.0%) than PC2 did (32%), while PC3 explained only 10.2% (Figure 4D; Table S4). In relation to PC1, nodule number (0.41), plant-N content (0.52), BNF potential (0.45), and RWC (−0.40) displayed the most variable values (Table S4). With regard to PC2, plant-P content (0.45) and Chl content (0.58) displayed the highest positive score (Table S4). Under WW and moderate available soil-Pi conditions, PC indicated 52.5 (PC1), 35.8 (PC2), and 6.0% (PC3) of data variability (Figure 4E; Table S4). Cowpea parameters shoot/root ratio (0.40), plant DM (0.40), plant-P content (0.42), RWC (0.44), and Chl content (0.45) displayed the highest positive score for PC1 (Table S4). On the other hand, plant-N (0.58) and BNF potential (0.57) exhibited the highest positive loading values for PC2 (Table S4). With the data obtained from plants grown under WW and high available soil-Pi supply, PC1, PC2, and PC3 explained 39.1, 29.6, and 11.0% data variability, respectively (Figure 4F; Table S4). Three out of the eight variables included in the analysis showed the highest positive values to PC1; specifically, plant DM (0.32), plant-N content (0.54), and BNF potential (0.30). With respect to PC2, variables with the highest positive or the lowest negative score were nodule number (−0.35), plant DM (0.49), plant-P content (0.60), plant RWC (−0.35), and Chl content (0.39) (Table S4).

## Discussion

WD and Pi deficiency are critical limiting abiotic stressors that negatively impact the nodulation, BNF, and thus legume growth and productivity worldwide (Sinclair et al., 2007; Tesfaye et al., 2007). To properly perform BNF under a combined stress of WD and Pi deficiency, legume plants should develop adequate mechanisms to efficiently use available soil-Pi, and to cope with WD, such as tolerance- and/or escape- and/or avoidance-related mechanisms (Vance, 2001; Sulieman et al., 2013; Nasr Esfahani et al., 2014; González et al., 2015). Cowpea plants grown in smallholder farms of the tropical countries should also fulfill these criteria to achieve the efficient BNF potential to satisfy their N demand, as well as the demand of other plants for an economic benefit and sustainability of N in cropping systems. Under the conditions of the smallholdings of the tropics that have erratic and limited rainfall conditions, many soils contain very low available soil-Pi (Fatokun et al., 2012; Jemo et al., 2015), thereby requiring frequent use of (in)-organic inputs to support crop growth (Manyong et al., 2001).

A number of research have taken the advantage of the rich genetic diversity of cowpea varieties, which are capable to confer numerous resistant traits to resist biotic and abiotic stresses, to increase cowpea grain yields (Daryanto et al., 2015; Boukar et al., 2016; Goufo et al., 2017; Huynh et al., 2017). However, for particular traits like BNF potential, which is an important agronomic trait when considering the legumes for intercropping and/or soil restoration, only limited scientific works have been conducted under individual WD or Pi deficiency stress, as well as under their combined stress (Daryanto et al., 2015; Abaidoo et al., 2017). Hence, in the present study, we used nine cowpea varieties of various origins (Table S1), and a comprehensive experimental approach to analyze the complex interactions between the SWC and soil-Pi availabilities with the ultimate aim to address two important objectives. First, we attempted to provide quantifiable agronomic data about the differential effects of SWC and available soil-Pi on nodulation, BNF potential and growth of cowpea plants under simulated conditions that are more or less similar to what cowpea crops often confront with in smallholdings in West African drylands. Second, from the in-depth analyses of our data, we would be able to select cowpea varieties with improved resistance to WD and/or low-Pi stress to recommend to the farmers in the region.

### Effects of SWC and available soil-Pi on biomass production of cowpea varieties

The combined effects of WD and Pi deficiency adversely affected the tested cowpea varieties with regard to their plant DM (Figures 1A,B). The gradual supply of available-Pi from different soils resulted in a proportional increase in DM accumulation under WD and WW conditions (Figures 1A,B). Positive correlation between the supply of Pi and crop growth has been demonstrated in many types of soils of the Nigerian Savanna (Nwoke et al., 2003; Pypers et al., 2006). At various soil-Pi supplies, remarkable differences in plant DM among the cowpea varieties were noticed under both WD and WW conditions (Figures 1A,B). These results imply that different cowpea varieties are likely to possess different strategies to adapt and/or adjust to different SWC and/or soil available-Pi levels to support their growth. The supply of sufficient levels of Pi to water-stressed cowpea plants reduced the damaging effects of WD on plant DM with great variations observed among varieties (Figures 1A,B). In support of our results, previous findings also reported that sufficient supply of Pi could reduce WD damage on various legume crops under field conditions (Gobarah et al., 2006; Gunes et al., 2006; Jin et al., 2006). It is worth noting that the soil samples that have high-Pi content also have higher concentrations of other nutrients (i.e., N, Ca and Mg) (Table S3), which might also contribute to the improved DM, as well as nodulation, plant-P and plant-N contents, and BNF potential of cowpea plants (Figure 3).

### Effects of SWC and available soil-Pi on uptake of Pi and N by cowpea varieties

On the basis of the results obtained from the present study, significant genetic differences observed among the 9 cowpea varieties in uptake of Pi and N under WD at all soil-Pi levels in comparison with respective WW control (Figure 2, Table 3). Water shortage resulted in significant decrease in uptake of Pi and N at any soil-Pi levels. Sufficient soil-Pi levels were observed to reduce the WD damage on uptake of Pi and N in comparison with moderate or low soil-Pi supply (Figure 2, Table 3). Under WW conditions, positive effects of available soil-Pi levels on uptake of Pi and N were noticed (Figure 2, Table 3), which is in agreement with previous findings reported for various crops (Vance et al., 2003; Jones et al., 2004; Jemo et al., 2006). The supply of Pi to cowpea roots improved the P nutrition status, and subsequently enhanced the capability of WD resistance in cowpea, which was either associated with an enhanced ability of the roots to search for water in soils, or an improved maintenance of water in the plant tissues (Garg et al., 2004; Jin et al., 2006). During WD, several cowpea varieties showed higher increase in uptake of Pi and N following the high soil-Pi supply as compared with the moderate or low soil-Pi supply (Figures S2B,C). In the context of the smallholding conditions of the drylands of tropical Africa, it was reported that the application of 20–30 kg P ha^−1^ in the form of mono superphosphate or triple superphosphate could maintain adequate uptake of Pi and N, allowing legume crops to achieve better yields under WD conditions (Pypers et al., 2006).

### Effects of SWC and available soil-Pi on nodulation of cowpea varieties

Importantly, significantly lower nodulation levels were observed under WD compared with WW conditions at different soil-Pi levels (Table 3). The poor nodulation observed under WD could be explained by the fact that rhizobia forming symbiotic association with legume roots are sensitive to both WD and Pi deficiency (Nasr Esfahani et al., 2014; Sulieman and Tran, 2015). Under such stressful conditions, the invading symbiotic bacteria usually lose their DNA during bacterial cell conjugation and suffer morphological changes, leading to decreases in infection and nodulation rates (Stouthamer and Kooijman, 1993; Sulieman and Tran, 2015). Consequently, under WD and Pi deficiency poor nodule development was observed, leading to decreased BNF rate and reduced DM (Figures 1, 3; Table 3), consequently low yield of cowpea, as also evidenced in many other legume crops (Sinclair et al., 2007; Charlson et al., 2009; Gil-Quintana et al., 2013; Sulieman et al., 2013; Cabeza et al., 2014; Nasr Esfahani et al., 2014). On the other hand, the fact that we could not observe any visible nodules in any cowpea varieties under WD at any soil-Pi levels after 28 days of WD exposure clearly indicated that cowpea nodules experienced severe damaging effects that might lead to desiccation-related senescence and decomposition of the majority of cowpea nodules, which had been formed before the induction of water stress. This finding was supported by the results of a previous investigation of cowpea growth and nodulation under WD conditions in the West African Savanna (Fatokun et al., 2012).

### Effects of SWC and available soil-Pi on BNF potential of cowpea varieties

The present study highlighted highly damaging effects of WD and Pi deficiency on cowpea BNF (Table 1). The BNF potential under the combined effects of WD and low available soil-Pi stresses was found to be low in cowpea varieties (Figure 3), which is supported by previous works in other legume crops, such as soybean (Chen et al., 2007) and peanut (Devi et al., 2013). The reduction of BNF in plants under WD and Pi-deficient stresses could be attributed to limited oxygen supply to nodules, nodule carbon (C) shortage, oxidative stress, and/or limited ability of nodules to export and translocation of fixed-N products to the shoots of the host plants (Devi et al., 2013; Sulieman and Tran, 2013; Sulieman et al., 2013; González et al., 2015). The observed genetic variation in BNF potential under WD and low-Pi conditions indicates the prospects of identifying cowpea varieties with relatively efficient BNF capacity, as previously reported in other legume crops, such as soybean (Ladrera et al., 2007; Sulieman et al., 2015) and peanut (Devi et al., 2013), for recommendation to farmers in the region or to breeders for further genetic improvement activities. Several other studies have also reported that differences in stress resistance of BNF among varieties are inversely correlated to plant ureide-N concentrations in legumes (Purcell et al., 2000; Sinclair et al., 2007; Charlson et al., 2009; Gil-Quintana et al., 2013). In legumes that utilize ureides to export BNF products the allantoate amidohydrolases hydrolyze the ureide allantoate to ureidoglycolate, enabling the plants to gain access to N (Werner et al., 2013; Coleto et al., 2014). High ureide-N accumulation is known to be associated to the rise in asparagine concentration, which inhibits the allantoate amidohydrolase function, leading to the observed accumulation of ureides under WD. This in turn may be one of the feedback signals for the shutdown of BNF activity (Werner et al., 2013). It will be important to further investigate whether ureide-N accumulation in response to WD and/or Pi deficiency in stems and/or nodules of sensitive cowpea varieties is also influenced by their rhizobial symbiotic counterpart.

### Selection of cowpea varieties with improved performance under WD and low soil-Pi conditions

Cowpea has an enormous ability to produce grains under magnitudes of WD that would render comparable crops unproductive (Fatokun et al., 2012; Goufo et al., 2017). Plants can alter their shoot/root ratio to properly adapt to various stressful conditions (Fenta et al., 2012; Durand et al., 2016; Kunert et al., 2016). However, the interactive effects of the stressful factors (e.g., WD and low-Pi levels) on the shoot/root ratio can be modulated by the combined actions of other related mechanisms/effects (Chaves et al., 2002; González et al., 2015). In the present study, the adjustment of shoot/root ratio could be negatively related; for instance, to the changes in Chl content and RWC, implying that various physiological mechanisms are likely involved in cowpea resistance to WD. As evidenced by PC analysis, the growth and BNF potential performance of varieties Dan'Ila and IT06-281-1 were correlated with PC1, while their Chl content and RWC were positively related to PC2 under WD and low available soil-Pi conditions (Figure 4A; Table S4). These data also suggest that Dan'Ila and IT06-281-1 might alter their carbohydrate metabolism and partitioning in leaves under stressful conditions, resulting in lower levels of C allocation to shoots and higher C accumulation in roots to maximize the root DM production (Hermans et al., 2006). Consequently, they reduce their shoot growth and conserve moisture in all plant tissues, thereby allowing adaptive adjustment of their shoot/root ratio for a better survival under stress. Furthermore, nodule N metabolism is closely connected with C metabolism in symbiotic plants, and a limitation in C supply will lead to reduced BNF capacity (Ladrera et al., 2007; Palma et al., 2013; Tsikou et al., 2013).

Results of present study firmly showed that varieties IT04K-339-1, IT07K-188-49, IT07K-304-9, and IT04K-405-5 exhibited greater performance in terms of plant growth, nodulation, plant biomass and BNF potential that were in the direction of the PC1 under low available soil-Pi and WD conditions (Figures 1–4; Table 3; Table S4). At the present stage of our investigation, this finding opens an opportunity to detect candidate genes that can be used to enhance traits associated with adaptation to WD and/or Pi deficiency. Various traits related to the adaptation of cowpeas in the tropics have been investigated and quantitatively mapped using quantitative trait locus (QTL) mapping approaches (Timko et al., 2007; Boukar et al., 2016; Huynh et al., 2017). In comparison with cowpea, breeding activities to improve the BNF traits is more advanced in other species, such as common bean (*Phaseolus vulgaris*) (Polania et al., 2016) and soybean (Muñoz et al., 2016), with the release of varieties with high BNF. As for cowpea, the availability of superior varieties with higher BNF abilities can offer multiple advantages to the farmers who have insufficient access to fertilizer inputs. An effective BNF from the cowpea will contribute to their own N need, as well as to satisfy the N requirement of other associated or intercropped non-legume crops (Udvardi and Poole, 2013).

In conclusion, the present work has allowed us to investigate the BNF capacity of cowpea varieties under WD and/or low soil-Pi conditions of the Nigerian Savanna. This is the first study to investigate how the interactions between WD and Pi deficiency adversely affect the BNF of cultivated cowpea plants in drylands of low available soil-Pi levels in West Africa. These results provide a basic foundation for selection of stress-resistant cowpea varieties that can be grown under extreme environmental conditions of smallholdings of West Africa or used in breeding research program for the benefit of the natives of the region. Among nine cowpea varieties examined in this study, varieties IT04K-339-1, IT07K-188-49, IT07K-304-9, and IT04K-405-5 were identified as the best performants in terms of nodulation, plant biomass production, uptake of N and Pi, and BNF potential under WD and Pi deficiency. These varieties could be used for further testing under field conditions, prior to recommending them for cultivating on low-Pi soils, drylands or drylands of low available soil-Pi levels in the region. These varieties can also be used as genetic resources for breeding activities. In addition, the BNF potential could be integrated among traits for selecting WD- and/or Pi deficiency-tolerant varieties, given that it is an important determining factor of yield potential. On the other hand, variety Dan'Ila displayed high capacity to physiological adjustment, in terms of shoot/root ratio and Chl content, to adapt to WD and Pi deficiency stresses. Taking advantage of the observed traits, further investigations leading to a better understanding of associated mechanisms will help develop variety(ies) of better BNF performance and physiological adjustment under WD and Pi-deficient conditions. Through these efforts, we aim to generate helpful information to support plant breeders in their efforts to develop high yielding and WD- and/or low available soil-Pi-adapted/resistant germplasm for the benefit of smallholder farmers of the dry savanna.

## Author contributions

MJ and OO: conceived and designed the experiments; OO and MJ: performed the experiments; MJ, OO, SS, FB, AH, EA, and AA: analyzed the data with the input from L-SPT; MJ and EA: contributed reagents, materials, analysis tools; MJ, OO, SS, FB, AH, EA, and L-SPT: wrote the paper.

### Conflict of interest statement

The authors declare that the research was conducted in the absence of any commercial or financial relationships that could be construed as a potential conflict of interest.
