# Supplementary material for: Comparative Analysis of the Combined Effects of Different Water and Phosphate Levels on Growth and Biological Nitrogen Fixation of Nine Cowpea Varieties
Source: Front Plant Sci. 2017 Dec 19;8:2111. doi: 10.3389/fpls.2017.02111 (PMC5742256; doi:10.3389/fpls.2017.02111)
Supplement: Supplementary file 1 [file Presentation1.pdf]

Supplementary Materials

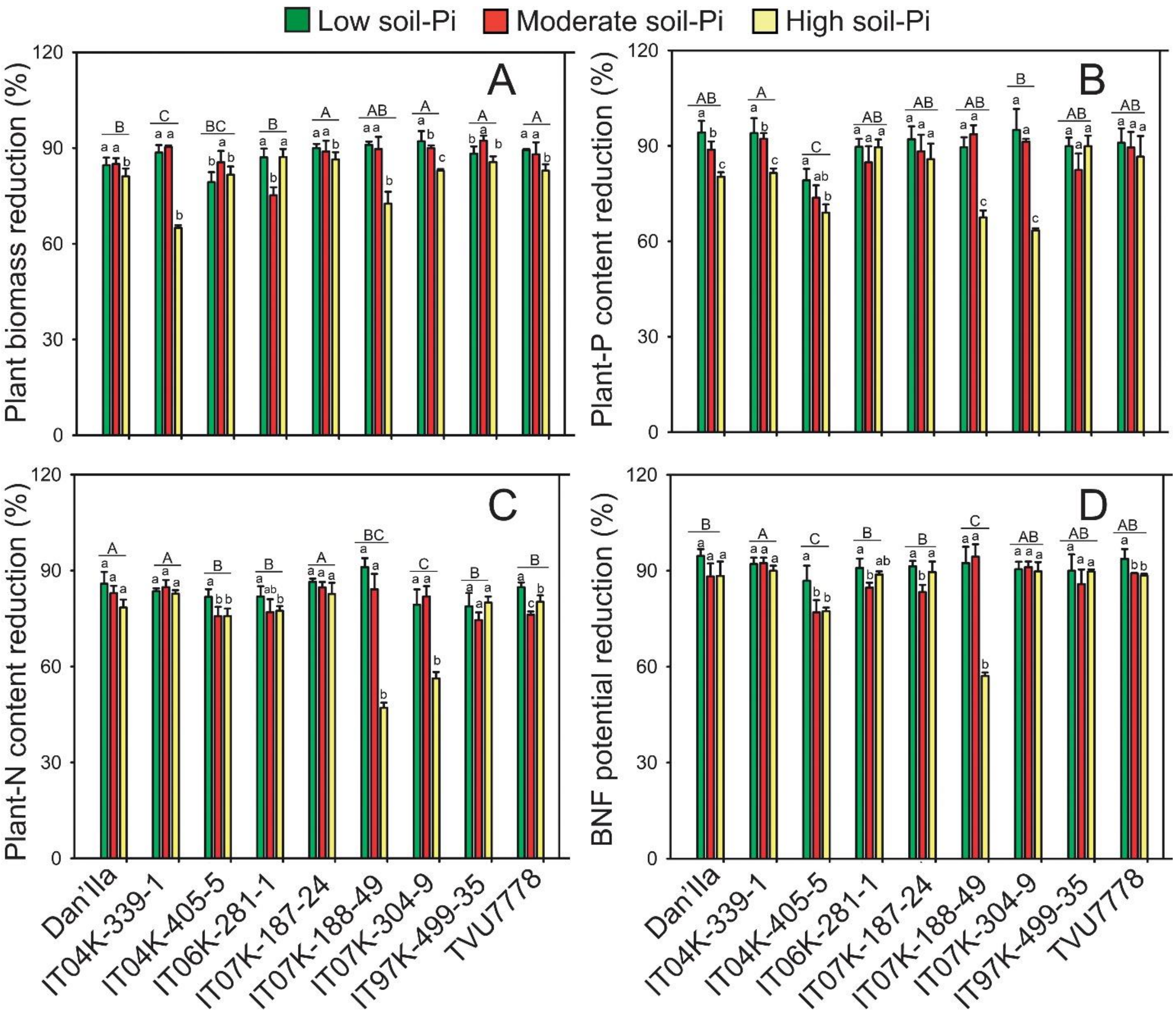

Figure S1. Water deficit reduced **(A)** plant biomass, **(B)** plant-P content, **(C)** plant-N content, and **(D)** biological nitrogen fixation (BNF) potential of cowpea varieties under different available soil-phosphate (Pi) levels in comparison with respective well-watered control. Mean values and standard errors (bars) are shown (n = 4). Different small letters indicate significant differences among the low, moderate and high available soil-Pi levels for each variety (Fisher's test; $p < 0.05$). Different capital letters indicate significant differences among the varieties using the combined data obtained from low, moderate and high available soil-Pi levels (Fisher's test; $p < 0.05$).

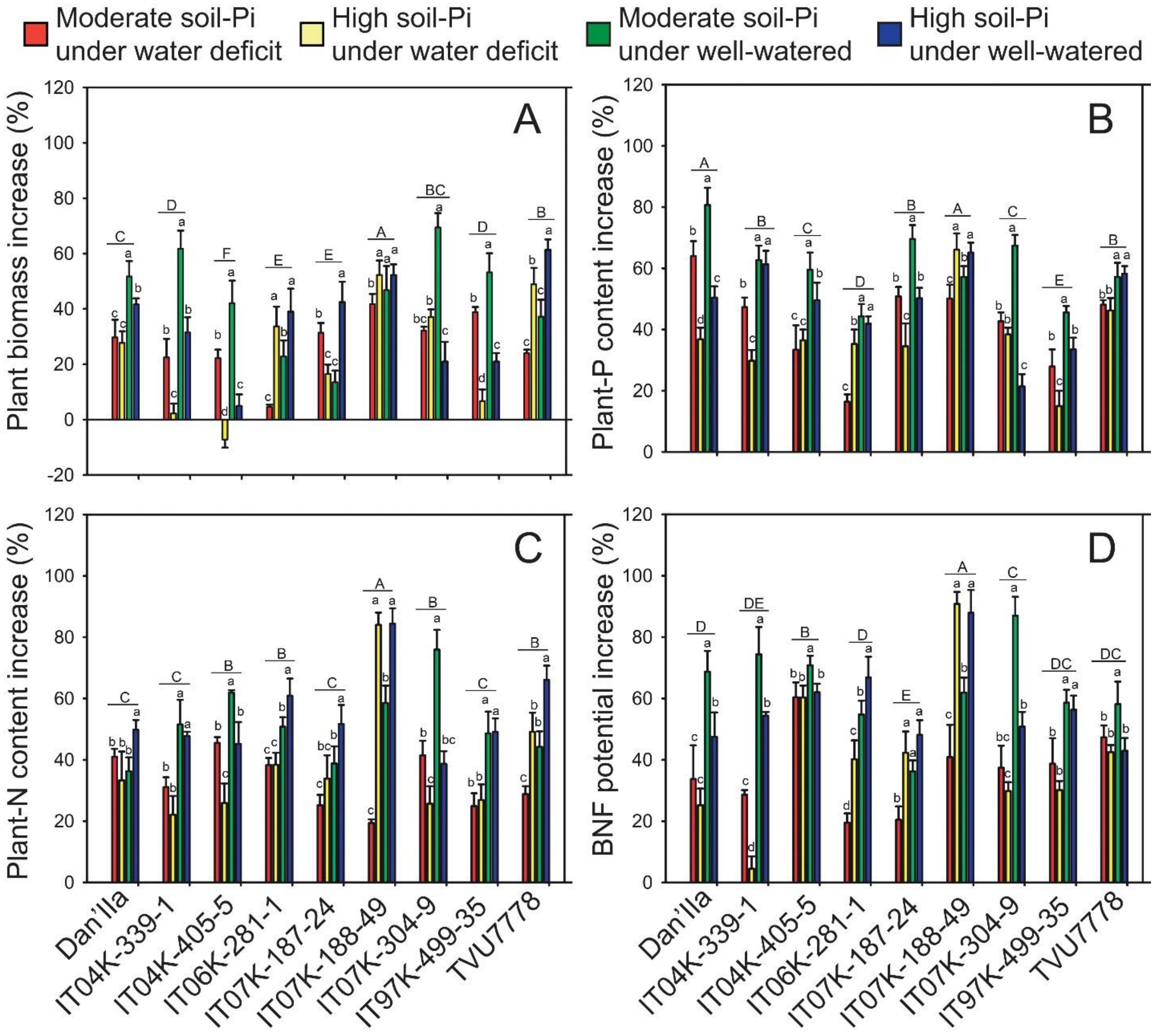

Figure S2. Available moderate and high soil-phosphate (Pi) levels increased **(A)** plant biomass, **(B)** plant-P content, **(C)** plant-N content, and **(D)** biological nitrogen fixation (BNF) potential of cowpea varieties under water deficit and well-watered conditions relative to that of plants grown on soils with low available-Pi levels under water deficit and well-watered conditions, respectively. Mean values and standard errors (bars) are shown (n = 4). Different small letters indicate significant differences among the growth conditions examined for each variety (Fisher's test; $p < 0.05$). Different capital letters indicate significant differences among the varieties using the combined data obtained from all the growth conditions examined (Fisher's test; $p < 0.05$).

TABLE S1. Growth characteristics and origin of the tested cowpea varieties

**Varieties**	**Growth season**	**Growth habit**	**Resistance to disease/parasitic attacks**	**Drought tolerance or susceptibility (for plant survival)**	**Origin**	**Seed characteristics**
IT07K-187-24	Early to medium (70 days)	Erect broad	Major diseases, striga	Unknown	Nigeria	White, smooth
IT06K-281-1	Medium (75-80 days)	Erect broad	Major diseases, striga	Unknown	Nigeria	White, smooth
IT07K-188-49	Early to medium (70 days)	Semi-spreading	Aphids, thrips, bruchids	Unknown	Nigeria, Kanananndo, Tanzania	White, rough
IT04K-339-1	Medium (80-85 days)	Very erect	Multiple diseases	Unknown	Nigeria, Tanzania	White, brown eye
IT07K-304-9	Medium (75 days)	Long peduncles, over canopy	Multiple diseases	Unknown	Nigeria, Tanzania, USA	White, rough
TVU7778	(75-80 days) (80-85 days)	Semi-erect	Aphids, thrips	Susceptible	Nigeria, Tanzania	Brown, rough
Dan'IIa	Late (80-85 days)	Spreading	Reduced disease attacks	Tolerant	Nigeria	White, rough
IT04K-405-5	Late (80-85 days)	Spreading	Reduced disease attacks	Unknown	Nigeria	Brown, rough
IT97K-499-35	Late (80-85 days)	Semi-erect	Aphids, thrips	Unknown	Nigeria, Tanzania	White, rough

TABLE S2. Field history with preceding crops at the sampled sites, location and selected chemical properties (topsoil 0–20 cm) of soil samples used in this study.

Sampled location	Field history	Soil-Pi level	Available-Pi [mg Pi kg^{-1}]	[1]pH	N content [mg N kg^{-1}]	Ca content [cmol kg^{-1}]	Mg content [cmol kg^{-1}]	Coordinates		Height above sea level
KARO	Forest	Low	1.13	6.1	300.6	1.95	0.09	N09 19.884'	E004 48.729'	182
EDUGI	Forest	Low	1.17	5.8	407.8	2.21	0.55	N09 19.678'	E005 13.938'	137
EDUGI	Groundnut	Low	1.31	5.5	224.2	1.28	0.74	N09 19.678'	E005 13.938'	137
KARO	Cowpea	Low	1.35	6.5	215.6	2.33	0.33	N09 19.884'	E004 48.729'	182
T/SABO TANAPA	Groundnut	Low	1.40	6.5	213.5	4.95	0.53	N09 10..248'	E005 11.391'	101
NDANAWUN	Forest	Low	1.43	6.2	320.5	1.78	0.44	N 09 08.358'	E 005 21.373'	115
EDUGI	Cowpea	Low	1.52	5.7	346.0	1.78	0.26	N09 19.678'	E005 13.938'	137
IITA MOKWA	Groundnut	Low	1.64	6.1	297.9	2.04	0.58	N 09 21.059'	E005 01.731'	175
SHETI	Cowpea	Low	1.67	6.2	420.5	2.42	0.49	N09 21.835'	E005 19.145'	179
KARO	Soybean	Low	1.80	6.5	284.4	2.10	0.33	N09 19.884'	E004 48.729'	182
SHETI	Forest	Low	1.96	5.8	473.4	2.33	0.31	N09 21.835'	E005 19.145'	179
DAKOGI	Groundnut	Low	1.96	7.6	376.9	3.58	0.37	N 09 09.896'	E 005 18.462'	159
TSAFA	Groundnut	Low	2.23	6.9	269.4	2.91	0.48	N09 13.333'	E004 52.766'	103
LAFIAGI	Groundnut	Low	2.31	6.2	333.0	1.28	0.58	N 09 15.354'	E 004 59.072'	149
KARO	Groundnut	Low	2.35	6.3	332.3	2.50	0.30	N09 19.884'	E004 48.729'	182
DAKOGI	Forest	Low	2.44	6.8	294.8	2.33	0.32	N 09 09.896'	E 005 18.462'	159
DAKOGI	Cowpea	Low	2.49	7.0	396.9	2.91	0.45	N 09 09.896'	E 005 18.462'	159
EDUGI	Soybean	Low	2.62	5.9	664.6	2.82	0.32	N09 18.896'	E005 15.210'	154
KOKODI	Cowpea	Low	2.65	7.0	1508.2	6.08	0.37	N09 13.333'	E 004 52.766'	115
IITA MOKWA	Cowpea	Low	2.77	6.9	465.1	3.49	0.30	N 09 21.059'	E005 01.731'	175
SHETI	Groundnut	Moderate	3.26	7.3	580.0	4.77	0.45	N09 21.835'	E005 19.145'	179
IITA MOKWA	Forest	Moderate	3.55	7.2	514.5	3.14	0.27	N 09 21.059'	E005 01.731'	175
TUNGA SALIHU	Forest	Moderate	3.57	7.3	428.5	3.70	0.20	N09 15.372'	E004 49.724'	156
LAFIAGI	Cowpea	Moderate	3.72	6.5	352.6	1.95	0.25	N 09 15.354'	E 004 59.072'	149
SHETI	Soybean	Moderate	3.83	6.5	744.5	3.29	0.40	N09 21.835'	E005 19.145'	179
KOKODI	Groundnut	Moderate	3.92	7.1	315.6	2.71	0.66	N09 13.333'	E 004 52.766'	115
TUNGA SALIHU	Soybean	Moderate	4.44	7.1	1011.6	8.12	0.43	N09 15.372'	E004 49.724'	156
TUNGA SALIHU	Cowpea	Moderate	5.15	7.4	359.9	2.91	0.50	N09 15.372'	E004 49.724'	156
NDANAWUN	Cowpea	Moderate	5.24	6.5	255.9	1.72	0.44	N 09 08.358'	E 005 21.373'	115
KOKODI	Soybean	Moderate	8.00	6.3	575.4	4.37	0.48	N09 13.333'	E 004 52.766'	115
TSAFA	Forest	Moderate	8.33	6.6	447.1	3.70	0.44	N09 13.333'	E004 52.766'	103
IITA MOKWA	Soybean	Moderate	8.63	6.1	206.3	1.25	0.13	N 09 21.059'	E005 01.731'	175

T/SABO TANAPA	Forest	Moderate	9.08	7.4	401.9	6.02	0.57	N09 10..248'	E005 11.391'	101
T/SABO TANAPA	Cowpea	High	10.30	6.6	240.9	2.50	0.74	N09 10..248'	E005 11.391'	101
NDANAWUN	Groundnut	High	10.58	6.9	435.4	3.49	0.29	N 09 08.358'	E 005 21.373'	115
TSAFA	Cowpea	High	13.30	8.0	457.0	5.91	0.38	N09 13.333'	E004 52.766'	103
TUNGA SALIHU	Groundnut	High	13.43	7.1	407.6	3.46	0.51	N09 15.372'	E004 49.724'	156
TSAFA	Soybean	High	13.88	6.6	735.6	3.29	0.70	N09 13.333'	E004 52.766'	103
KOKODI	Forest	High	14.43	7.4	777.0	6.98	0.52	N09 13.333'	E 004 52.766'	115
LAFIAGI	Forest	High	17.87	6.6	896.1	3.35	0.20	N 09 15.354'	E 004 59.072'	149
T/SABO TANAPA	Soybean	High	28.28	6.7	574.4	4.77	0.41	N09 10..248'	E005 11.391'	101

[1]pH measured in water.

TABLE S3. Soil-pH, and -Pi, -N, -Ca and -Mg concentrations in the topsoil (0–20 cm) collected from the experimental sites. Mean values ± standard errors are shown (n ≥ 8 field samples/soil-Pi level). Different letters indicate significant differences ($p < 0.05$) between the low, moderate and high available soil-phosphate (Pi) levels (Fisher's test; $p < 0.05$).

Soil-Pi level	Number of sampled fields	Available soil-Pi [mg Pi kg^{-1}]	pH[1]	N concentration [mg N kg^{-1}]	Ca concentration [cmol kg^{-1}]	Mg concentration [cmol kg^{-1}]	Height above sea level [m]
Low	20	1.9 ± 0.11 c	6.4 ± 0.12 b	407± 61.0 b	2.65 ± 0.25 c	0.41± 0.03 b	153
Moderate	13	5.4 ± 0.6 b	6.8 ± 0.12 a	476.5± 60.2 b	3.7± 0.51 b	0.40± 0.04 b	144
High	8	15.3 ± 2.0 a	6.9 ± 0.18 a	565± 78.6 a	4.8± 0.54 a	0.50± 0.07 a	177

[1]pH measured in water

TABLE S4. Principal component (PC) scores of the cowpea parameters displaying the loadings from each included parameter for the water deficit and low available soil-Pi, water deficit and moderate available soil-Pi, water deficit and high available soil-Pi, well-watered and low available soil-Pi, well-watered and moderate available soil-P, and well-watered and high available soil-Pi conditions.

	PC1	PC2	PC3	PC4	PC5	PC1	PC2	PC3	PC4	PC5
	Water deficit and low available soil-Pi level					Well-watered and low available soil-Pi level				
Nodule number (plant^{-1})	0.38	-0.40	0.09	0.05	0.70	0.41	-0.30	0.06	-0.27	0.55
Shoot root ratio [(g shoot DW)/(g root DW)]	0.07	0.61	-0.01	0.70	0.06	-0.20	-0.23	0.93	0.11	-0.07
Plant (shoot + root) biomass DM [g plant^{-1}]	0.50	0.11	0.04	0.00	-0.25	0.26	0.42	0.30	-0.55	-0.21
Plant (shoot + root) P content [mg P plant^{-1}]	0.39	-0.26	0.40	0.33	-0.21	0.27	0.45	0.05	0.46	-0.37
Plant (shoot + root) N content [mg N plant^{-1}]	0.45	-0.09	-0.26	0.16	-0.01	0.52	0.10	0.06	-0.08	0.02
BNF potential [mg N plant^{-1}]	0.42	0.07	-0.39	-0.35	-0.39	0.45	0.01	0.15	0.56	0.34
Plant relative water content [%]	0.10	0.21	0.78	-0.30	-0.14	-0.40	0.36	0.03	0.20	0.49
Chlorophyll content [µmol per m^2 of leaf surface]	0.25	0.57	-0.03	-0.39	0.47	-0.12	0.58	0.12	-0.19	0.39
Eigen value	3.8	1.8	1.3	0.6	0.3	3.6	2.6	0.8	0.7	0.3
Variance explained (%)	47.2	22.7	16.8	7.3	3.5	45.0	32.0	10.2	8.8	3.3
	Water deficit and moderate available soil-Pi level					Well-watered and moderate available soil-Pi level				
Nodule number (plant^{-1})	0.17	-0.39	0.57	-0.40	0.11	-0.31	0.36	0.40	0.63	0.02
Shoot root ratio [(g shoot DW)/(g root DW)]	-0.10	-0.08	0.67	0.55	-0.40	0.40	-0.11	0.71	-0.34	-0.10
Plant (shoot + root) biomass DM [g plant^{-1}]	0.52	-0.05	-0.06	-0.12	-0.39	0.40	0.29	0.15	0.41	-0.39
Plant (shoot + root) P content [mg P plant^{-1}]	0.51	-0.01	-0.05	-0.26	-0.26	0.42	0.26	-0.28	-0.14	-0.24
Plant (shoot + root) N content [mg N plant^{-1}]	0.40	0.29	0.02	0.49	0.46	0.03	0.58	-0.20	-0.14	0.48
BNF potential [mg N plant^{-1}]	0.45	0.36	0.11	0.13	-0.01	0.08	0.57	0.15	-0.32	0.04
Plant relative water content [%]	-0.07	0.51	0.45	-0.42	0.36	0.44	-0.21	0.15	0.26	0.74
Chlorophyll content [µmol per m^2 of leaf surface]	0.24	-0.61	0.01	0.15	0.52	0.45	-0.09	-0.38	0.33	-0.09
Eigen value	3.4	1.8	1.3	1.0	0.3	4.2	2.9	0.5	0.3	0.1
Variance explained (%)	42.4	23.1	16.4	12.7	3.6	52.5	35.8	6.0	4.3	0.9
	Water deficit and high available soil-Pi level					Well-watered and high available soil-Pi level				
Nodule number (plant^{-1})	-0.04	0.43	0.57	0.54	0.41	0.19	-0.35	0.59	-0.28	0.24
Shoot root ratio [(g shoot DW)/(g root DW)]	0.43	-0.22	0.33	0.11	-0.44	-0.50	0.04	0.21	0.38	0.39
Plant (shoot + root) biomass DM [g plant^{-1}]	0.47	0.20	-0.10	-0.21	0.29	0.32	0.49	-0.14	0.15	0.53
Plant (shoot + root) P content [mg P plant^{-1}]	0.22	-0.11	-0.68	0.59	0.14	0.17	0.60	-0.01	-0.04	-0.43
Plant (shoot + root) N content [mg N plant^{-1}]	0.48	-0.14	0.19	-0.25	0.03	0.54	0.07	0.10	-0.14	0.39
BNF potential [mg N plant^{-1}]	0.47	0.19	-0.09	-0.15	0.35	0.30	-0.03	0.46	0.76	-0.26
Plant relative water content [%]	0.19	-0.61	0.23	0.40	0.05	0.13	-0.35	-0.59	0.39	0.20
Chlorophyll content [µmol per m^2 of leaf surface]	0.24	0.55	-0.08	0.25	-0.64	-0.42	0.39	0.17	0.00	0.25
Eigen value	3.7	1.8	1.0	0.9	0.5	3.12	2.4	1.5	0.7	0.2

Variance explained (%)	45.9	22	12.9	11.3	6.0	45.0	32.0	11.0	4.4	1.6

Data of nodules number were log (x+1)-transformed. Data of plant relative water content (percentage values) were arsine square-root transformed. Data of the plant biomass, plant-P and -N contents and biological nitrogen fixation (BNF) potential were square root-transformed before being included into the analysis. DM, dry matter.
